# The impact of the COVID-19 pandemic on the medical care and health-care behaviour of patients with lupus and other systemic autoimmune diseases: a mixed methods longitudinal study

**DOI:** 10.1093/rap/rkaa072

**Published:** 2020-12-14

**Authors:** Melanie Sloan, Caroline Gordon, Rupert Harwood, Elliott Lever, Chris Wincup, Michael Bosley, James Brimicombe, Mark Pilling, Stephen Sutton, Lynn Holloway, David D’Cruz

**Affiliations:** Department of Public Health and Primary Care, School of Clinical Medicine, University of Cambridge, Cambridge; Rheumatology Research Group, Institute of Inflammation and Ageing, College of Medical and Dental Science, University of Birmingham, Birmingham; Patient and Public Involvement in Lupus Research Group, Institute of Public Health, University of Cambridge, Cambridge; Rheumatology Department, University College London Hospital; Rheumatology Department, University College London Hospital; Patient and Public Involvement in Lupus Research Group, Institute of Public Health, University of Cambridge, Cambridge; Department of Public Health and Primary Care, School of Clinical Medicine, University of Cambridge, Cambridge; Department of Public Health and Primary Care, School of Clinical Medicine, University of Cambridge, Cambridge; Department of Public Health and Primary Care, School of Clinical Medicine, University of Cambridge, Cambridge; Patient and Public Involvement in Lupus Research Group, Institute of Public Health, University of Cambridge, Cambridge; Louise Coote Lupus Unit, Guys’ and St Thomas’ NHS foundation Trust, London, UK

**Keywords:** rheumatology, COVID-19, pandemic, SLE, lupus, systemic autoimmune rheumatic diseases, patient behaviour, psychology, patient care

## Abstract

**Objective:**

The aim was to explore the self-reported impact of the COVID-19 pandemic on changes to care and behaviour in UK patients with systemic autoimmune rheumatic diseases, to help ensure that patient experiences are considered in future pandemic planning.

**Methods:**

This was a longitudinal mixed methods study, with a cohort completing baseline surveys in March 2020 and follow-up surveys in June 2020 (*n* = 111), combined with thematic analysis of the LUPUS UK forum and participant interviews (*n* = 28).

**Results:**

Cancellations of routine care and difficulties in accessing medical support contributed to some participants deteriorating physically, including reports of hospitalizations. The majority of participants reported that fear of COVID-19 and disruptions to their medical care had also adversely impacted their mental health. Feeling medically supported during the pandemic was correlated with multiple measures of mental health and perceptions of care, including the Warwick–Edinburgh mental well-being score (*r* = 0.44, *P* = 0.01). Five themes were identified: detrimental reduction in care; disparities in contact and communication (medical security *vs* abandonment sub-theme); perceived and actual endangerment; the perfect storm of reduced clinician ability to help and increased patient reticence to seek help; and identifying the patients most vulnerable to reduced medical care.

**Conclusion:**

The diversion of resources away from chronic disease care was perceived by many participants to have caused adverse outcomes. Fear about increased vulnerability to COVID-19 was high, contributing to health-care-avoidant behaviours. This study also highlights the influence of clinician accessibility and patients feeling medically supported on multiple measures of physical and mental health.

**Key messages**

- Diversion of resources to COVID-19 was considered to contribute to multiple adverse outcomes, including hospitalizations.
- Findings highlight the need for rheumatology departments to implement robust procedures to ensure continued access to services.
- Feeling medically supported was positively associated with overall well-being scores during the COVID-19 pandemic.

## Introduction

The National Health Service (NHS) was already in a weakened state [1] and the UK unprepared for a pandemic [2] when the first UK coronavirus disease (COVID-19) case was recorded in January 2020 [3]. Less than 2 months later, hospitals were instructed to postpone non-urgent care [4], and visits to general practitioner (GP) practices [5] and accident and emergency (A&E) departments also declined [4]. These changes disrupted the provision of care for patients with chronic illnesses [6], and there are indications that this could have impacted those with lupus and other systemic autoimmune rheumatic diseases (SARDs) disproportionately. This was attributable, in part, to rheumatologists having been largely redeployed to the COVID-19 front line [7], where they were uniquely qualified to help manage the potentially fatal hyperinflammatory complications of COVID-19 [8], with other rheumatology staff also being redeployed [9].

The consequent reduction in usual rheumatology care was of particular concern, in that SARD patients often have complex multi-system disease with potentially life-threatening manifestations [10], which are, in general, poorly understood by non-specialist clinicians [11, 12]. It is of paramount importance to ensure that these SARD manifestations and ‘undetected serious adverse effects of ongoing treatments’ [13], particularly immunosuppressants [10], do not lead to future complications. In addition, medical insecurity and distrust among SARD patients who experienced traumatic diagnostic journeys and previous negative medical interactions [14] could have left them psychologically vulnerable to COVID-19-related reductions in quality and continuity of care. Further distress might have arisen from uncertainty about whether immunosuppressive medication increased or reduced the risk of severe COVID-19 [15], along with concerns about the availability of hydroxychloroquine [16, 17] and from inadequate information about their own individual COVID-19-related risk, with a recent paper suggesting that at least one-third of SLE patients should have been advised to shield [18].

The principal aim of this study was to understand more fully the impact of these COVID-19-related changes in health care, in order that the health services are better able to deliver effective care for SARD patients during the continuing pandemic [19] and to plan for future UK health crises. In so planning, it is essential to ensure that the experiences and views of patients are heard and acted upon, particularly with regard to understanding the effects of feeling medically supported (or not) during times of personal and national health crises.

## Methods

### Data collection

This was a sequential multi-phase mixed methods design, with the qualitative components being both exploratory and explanatory [20]. Ethical approval was obtained through the Cambridge Psychology Research Committee for each part of the research. Participants were recruited online through the LUPUS UK forum (>25 000 members) and Lupus support UK Facebook group (>7000 members) for a longitudinal pre-registered (ISRCTN-14966097) randomized controlled study investigating the feasibility and acceptability of peer support by small email groups. Fully informed consent was obtained online before questionnaire completion and recorded verbally before interviews. Inclusion criteria were as follows: a diagnosis of lupus or other SARD (as detailed on their clinic letters), ≥18 years of age and resident in the UK. The sample was based on opportunistic considerations.

Although the peer-support trial recruitment was coincidental to the pandemic, it provided a unique opportunity to compare the views of these patients at key stages of the pandemic. The surveys incorporated validated tools for assessing patient well-being, mental health and disease acceptance, including the Warwick–Edinburgh mental well-being scale (WEMWS) [21] and questions compiled by the study team and patients to capture perceived changes in care, medical support and impact on mental health owing to the pandemic. These were both positively and negatively framed to reduce bias. For example, ‘My doctors have helped me with my lupus/CTD symptoms’ and ‘My medical appointments for my lupus/CTD have been cancelled/postponed’. (Questions in this section were preceded with ‘During the coronavirus pandemic’, and response options were on a five-point scale of: much less, less, no change, more, much more.)

The study research stages and related key pandemic developments were as follows:

- Pre-lockdown online baseline survey (completed 4–10 March 2020, *n* = 139). World Health Organization (WHO) declared a pandemic (11 March 2020) and UK lockdown commenced (23 March 2020).
- Weekly email communications with groups of study participants on the impact of the pandemic, and ethnographic research within the LUPUS UK forum (March–July 2020). Staggered easing of lockdown across the four UK nations (May–July 2020).
- Follow-up survey (completed 10–21 June, *n* = 111).
- In-depth patient interviews (July 2020, *n* = 28). Shielding for at-risk groups ended (August 2020).

Interviewees were sampled purposively from follow-up survey responses to include a range of sociodemographic characteristics and medical experiences. Interview guides (Supplementary Data S1, available at *Rheumatology Advances in Practice* online) contained standardized and personalized questions designed from each survey response to explore patients’ views and experiences of medical care during the pandemic further. Interviews were conducted by M.S., mainly via the telephone, audio-recorded and transcribed verbatim, and lasted ∼60 min. Interviewing continued until thematic/theoretical saturation was reached, with this being taken to the point at which the themes together addressed all the main research questions and additional interviews did not provide significant new relevant insights or contradict the conclusions drawn from earlier interviews.

### Analysis

The integration of qualitative and quantitative data occurred at all stages. Analysis was thematic [22]; with M.S. using Nvivo12 to code qualitative data, and R.H. double-coding 25% of transcripts to test and enhance reliability. Potential threats to validity were addressed by ‘member checking’ [23], triangulation of quantitative and qualitative data and examination of cases that deviated from the norm [24]. Common themes and concepts emerging from the data were then discussed and agreed by the wider team and multiple patients. Quantitative data were analysed principally using Pearson’s correlation coefficient. More detailed methodology and the consolidated criteria for reporting qualitative research (COREQ) checklist [25] are included in Supplementary Data S2, available at *Rheumatology Advances in Practice* online.

## Results

The response rate for the surveys was 80% (111 of 139 completing follow-up). The majority of respondents were female (in keeping with lupus predominantly affecting women) and included a wide range of sociodemographic and disease characteristics, as shown in Table 1. Approximately 50% of survey participants reported being allocated to the shielding group.

**Table 1:** Table of participants: *n* = 111 (survey) *n* = 28 (interview)

Characteristic	Number (survey)	Percentage (survey)	Number (interview)	Percentage (interview)
**Age band, years**				
20–29	20	18	5	18
30–39	18	16	3	11
40–49	23	21	7	25
50–59	31	28	5	18
60–69	15	14	6	21
70+	4	4	2	7
**Diagnosis**				
SLE	87	78	17	61
UCTD	7	6	4	14
SS	5	5	2	7
MCTD or overlap CTD	6	5	2	7
Cutaneous lupus	4	4	2	7
Probable or incomplete lupus	2	2	1	4
**Employment**				
Employed full time	27	24	7	25
Employed part time	22	20	5	18
Self-employed	7	6	4	14
Not currently working owing to health	31	28	7	25
Retired	19	17	5	18
Other	5	5	0	0
**Ethnicity**				
Asian	6	5	2	7
White	100	90	22	79
Black	2	2	2	7
Mixed race	3	3	2	7
**Gender**				
Female	109	98	28	100
Male	2	2	0	0
**Qualifications**				
None	2	2	0	0
GCSE/O levels (equivalent)	19	17	4	14
A levels (or equivalent)	25	23	6	21
Degree or above	60	54	18	64
Prefer not to say	5	5	0	0
**Country of residence**				
England	84	76	20	71
Scotland	16	14	5	18
Wales	9	8	3	11
Northern Ireland	2	2	0	0
**Time since diagnosis, years**				
<1	10	9	5	18
1–2	16	14	3	11
3–5	21	19	3	11
6–10	32	29	7	25
11–20	18	16	8	29
>20	14	13	2	7

Themes arising from the qualitative data were as follows: detrimental reduction in care; disparities in contact and communication, incorporating a sub-theme of medical security *vs* abandonment; perceived and actual endangerment; the perfect storm of reduced clinician ability to help and increased patient reticence to seek help; and identifying the patients most vulnerable to reduced medical care.

Where percentages or any quantitative measures are given and/or respondents referred to, these are data from the survey, as opposed to interviewee data.

### Theme 1: Detrimental reduction in care

**Fig. 1:**
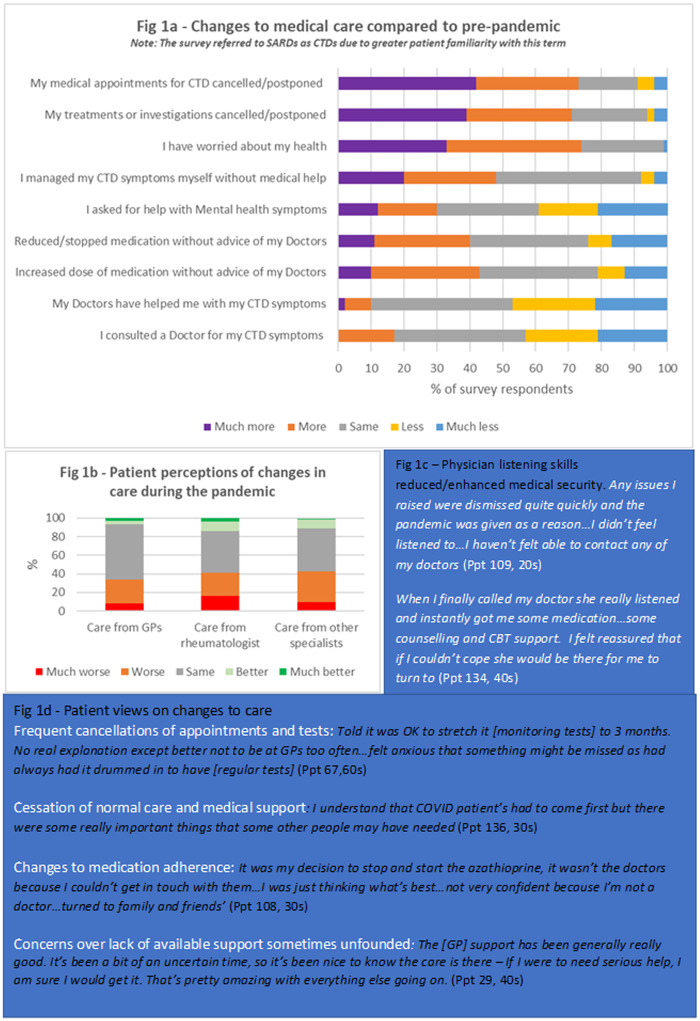
Perceptions of changes to care from pre-pandemic to during pandemic

Survey and interview results indicated significant detrimental changes in medical care for the majority of these patients, as shown in Fig. 1. More than 70% of respondents reported that their appointments, tests and treatments had been cancelled more/much more frequently since the pandemic began, and great concern was expressed about the lack of clinician availability when seeking support. Between 35 and 45% of respondents felt that care from GPs, rheumatologists and other specialists had been worse/much worse, and <30% reported feeling medically supported for their lupus/SARD during the pandemic.

**Fig. 2:**
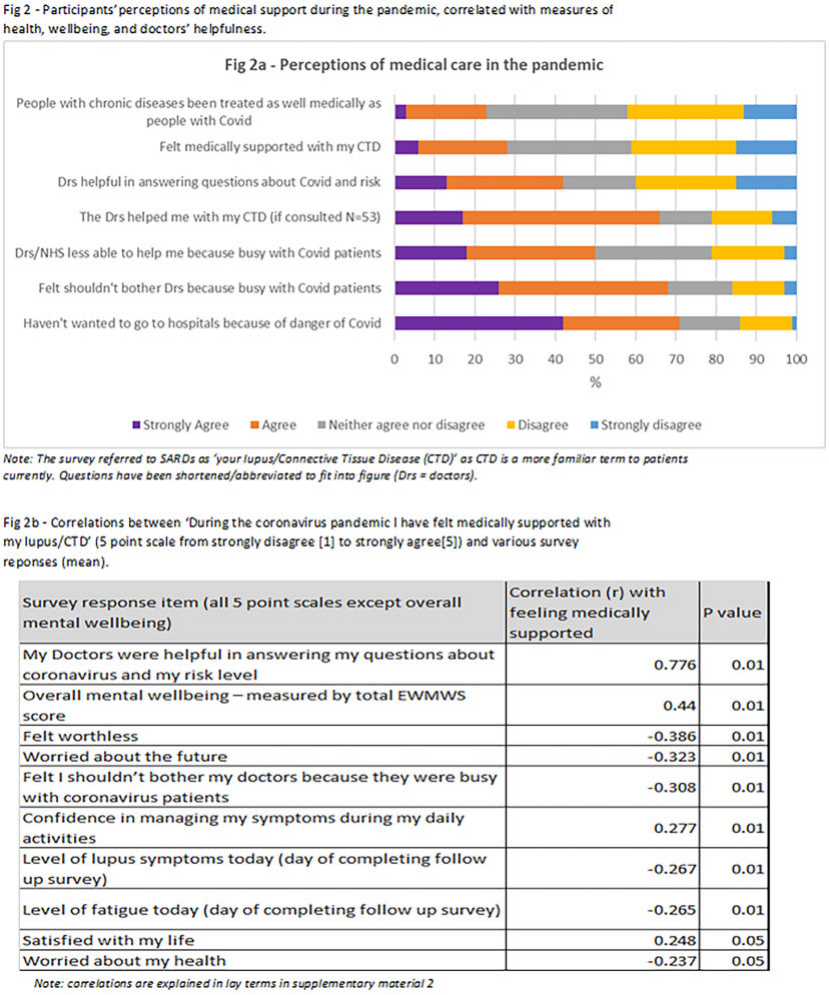
Participants’ perceptions of medical support during the pandemic, correlated with measures of health, well-being and doctors’ helpfulness

As shown in Fig. 2, patients reporting having felt medically supported was associated with overall reported well-being (*r* = 0.44, *P* = 0.01), measured by the validated WEMWS tool at baseline and follow-up, and was strongly correlated (*r* = 0.776, *P* = 0.01) with helpfulness of doctors in answering questions about COVID-19 and risk levels. It was also significantly, albeit weakly, correlated with multiple improvements to physical health, self-efficacy, fatigue and anxiety. However, there was no correlation with changes to medication adherence or in raising mental health concerns with physicians. Although participants discussed many negative impacts on their mental health from COVID-19, the response of the government to the pandemic and the reduction in medical care, no significant difference in overall well-being was measured on the WEMWS between baseline and follow-up.

### Theme 2: Disparities in contact and communication, incorporating a sub-theme of medical security *vs* abandonment

There was considerable variation in participant reports of the quality of contact and care. Explicit reassurance of physician accessibility and/or regular physician check-ins contributed to feeling cared about and medically secure:

I was given [contact details of specialist]. Reassurance. It gives me confidence to self-manage it because I know I can go to the team if I need to. (Participant 122, 60s)

It also allowed the physicians to identify declining mental health in addition to physical health issues and to keep communication channels open. Unsolicited contact was particularly appreciated:

My GP rang me a few times just to see how I was getting on … really nice … talked about mentally and physically … good to vent … said if I need anything to call, and I will. (Participant 75, 40s)

At the other end of the spectrum, there was a widespread sense of abandonment, especially among those whose routine care was abruptly stopped and who could not access assistance in an emergency. Multiple patients, both in the study and on the forum, detailed repeated unsuccessful attempts to contact their rheumatology department:

The system abandoned its existing patients to make room for new ones due to COVID-19. There are no longer people out there whom we can speak to and ask help. Which doctor is there to help me? I left three messages … emails … phonecalls … did not hear anything back … it is very easy to fall into depression. (Forum member, 40s)

Table 2 sets out suggestions to improve care and communication, along with illustrative quotations from patients. These suggestions are derived from an analysis of the relative success of the reported care and communication strategies and from direct suggestions from participants regarding what they felt would help in the future.

**Table 2:** Suggestions and quotations concerning disparities in care and communication

Suggestion	Positive patient experience	Negative patient experience
All patients should be provided with contact details of the rheumatology department and reassurance of prompt support	I know I can get hold of my rheumatologist if I need to, and she’s good at responding to emails and helping … makes me feel safer, erm, and it makes me have confidence in the health service and makes me worry less. (Participant 80, 20s)	I delayed contacting rheumatology as the letter I received said my appointment was not going ahead due to the pandemic and only to contact if I felt it was urgent. I carried on until my quality of life was unsustainable both physically and mentally … very depressed. (Participant 134, 40s)
Physicians should build trusting relationships with patients who have chronic diseases, and get to know them and their individual manifestations	[GP] who’s been there throughout with my lupus and she very much understands it, it was much easier to talk about these things, which very much helped with calming down the anxiety. (Participant 47, 50s)	I have had no contact from the GP or rheumatology. I already knew they didn’t care, but this just highlighted the fact.… Twice I have left messages and still no-one rings back, so I just manage … felt very alone and isolated. (Participant 132, 40s)
All patients should be contacted by a qualified clinician (preferably their own) to discuss individual risk levels	It felt very good to hear their advice about risk.… I feel glad to have specific personal advice from my consultant, who can weigh up my particular situation. Obviously, I have less confidence in generic government advice and its concern to balance economics and health. (Participant 105, 50s)	I have had no contact at all from my doctors regarding shielding. I spoke to my GP … seemed surprised that I wasn’t shielding … no more said. I was disappointed … concerned and confused about what I should be doing.… Some contact from them early on … advice and clarify their position would have been really helpful. (Participant 109, 20s)
Provision of prompt GP and specialist (within 24 h) response for all SARD patients owing to potential for rapid deterioration	I really like my GP … usually get him on the same day … advocates for me quite a lot, so when I was having problems right at the beginning of coronavirus … [GP] got that sorted out … was running out of methotrexate and couldn’t get it from the hospital, so he’s been very supportive.… (Participant 12, 40s)	All this stuff about the NHS is open, which has been the trope all the way through, I don’t buy, I really don’t, it hasn’t been.… Access to primary care has been really difficult … and [rheumatology] I think it has just stopped.… I’ve had new symptoms and I’m thinking what’s the point, I can’t get an appointment. (Participant 10, 60s)
Unsolicited contact by clinicians to check on physical and mental health	GP surgery called me a few times just to check up on me and see how I was doing and feeling.… Rheumatologist called … info. on shielding and see how I was doing … very helpful and kind.… I was very happy and felt that they cared. (Participant 136, 30s)	No one contacted me unless I called them first. I felt very unsupported and frightened through lockdown as I was suddenly 100% responsible for managing my lupus and other illnesses with no easy/safe access to medical care.… It’s as though I don’t exist. I have no idea how to keep myself and my family safe. (Participant 55, 40s)

GP: general practitioner; NHS: National Health Service; SARD: systemic autoimmune rheumatic disease.

The experiences detailed in the interviews and summarized in Table 2 highlight the importance of building a trusting medical relationship. Those with pre-existing secure medical relationships often expressed lower anxiety and more confidence that support would be forthcoming if required:

I feel they [GP and rheumatologist] care about me. More so my GP, who knows and understands my conditions.…When I was in lockdown, I knew I could call on him … took away a lot of the stress.… (Participant 124, 70s)

Interviews with other participants revealed discomfort and guilt from the difficulties in rationalizing the cognitive dissonance created by a feeling of personal medical abandonment, while also having concern for patients with COVID-19 and empathy for the pressures faced by their clinicians:

When in pain I feel as if my situation does not matter. [They are] obviously concentrating on looking after sick COVID patients.… I feel selfish for wishing my consultant was treating me and not them. I have seen the programmes on TV showing the stresses that front-line staff have been under. (Participant 67, 60s)

### Theme 3: Perceived and actual endangerment

Participants expressed a strong sense of being endangered from both the virus and the redeployment of medical resources. COVID-19 fears were often initiated or reinforced by strongly worded risk guidance from the government and physicians:

[Rheumatologist] said, ‘if you come to the hospital and get it, it's game over for you’. (Participant 79, 40s)

The repeated government warnings concerning the potential for the NHS to be overwhelmed increased both health-care avoidance and fears:

‘You’re going to have to look after yourself and keep as well as possible and avoid A&E or need health care because it’s not necessarily going to be available’ … definitely increased my anxiety. (Participant 108, 30s)

Although a majority of participants felt that their chronic disease care had been adversely impacted owing to prioritization of patients with COVID-19 (only ∼20% agreed that patients with chronic diseases had been treated as well as COVID-19 patients), some also feared that their care, and potentially their lives, would also be less valued if they contracted COVID-19:

Are we considered a class of patients that can be sacrificed for the good of healthy others? … Feel quite scared that I will be seen as less worthy of saving than someone else due to my autoimmune illnesses. (Participant 55, 40s)

Some participants discussed how their initial fears were sometimes unfounded. A high proportion (∼70%) of those who managed to access care reported strongly agreeing/agreeing that they had been helped with their SARD symptoms. Those who had been reticent to seek help owing to COVID-19 infection risk in health-care settings sometimes also reported that the high level of precautions created a greater feeling of safety than anticipated.

The lower numbers of the general population attending hospital also resulted in many more positive experiences owing to a less chaotic environment than normal, with likely COVID-19 patients often kept separate. However, with many rheumatologists redeployed, GPs and Emergency Medicine staff (although generally reported to be accessible and well-meaning) were strongly felt to lack sufficient knowledge of complex autoimmunity to manage these patients safely without specialist support:

[A&E] were good in that they were quick, but I wouldn’t say they were experts in lupus, or the medication … that was quite scary. (Participant 108, 30s)

Although many patients discussed feeling extremely endangered by COVID-19, few reported having COVID-19 symptoms (12%), and no survey respondents tested positive or were hospitalized for COVID-19 (although the avoidant behaviour will have reduced transmission likelihood). However, there were many reports of adverse impacts on physical and mental health from the reduction in care, including participants reporting attendance at A&E departments or being admitted, owing to untreated and/or uncontrolled disease activity or infections. Several participants felt this might have been avoidable and had tried unsuccessfully to contact rheumatology departments before deteriorating to the point of requiring admission. Others considered that the stress and lack of medical and/or government support had directly caused a flare.

### Theme 4: The perfect storm

**Fig. 3:**
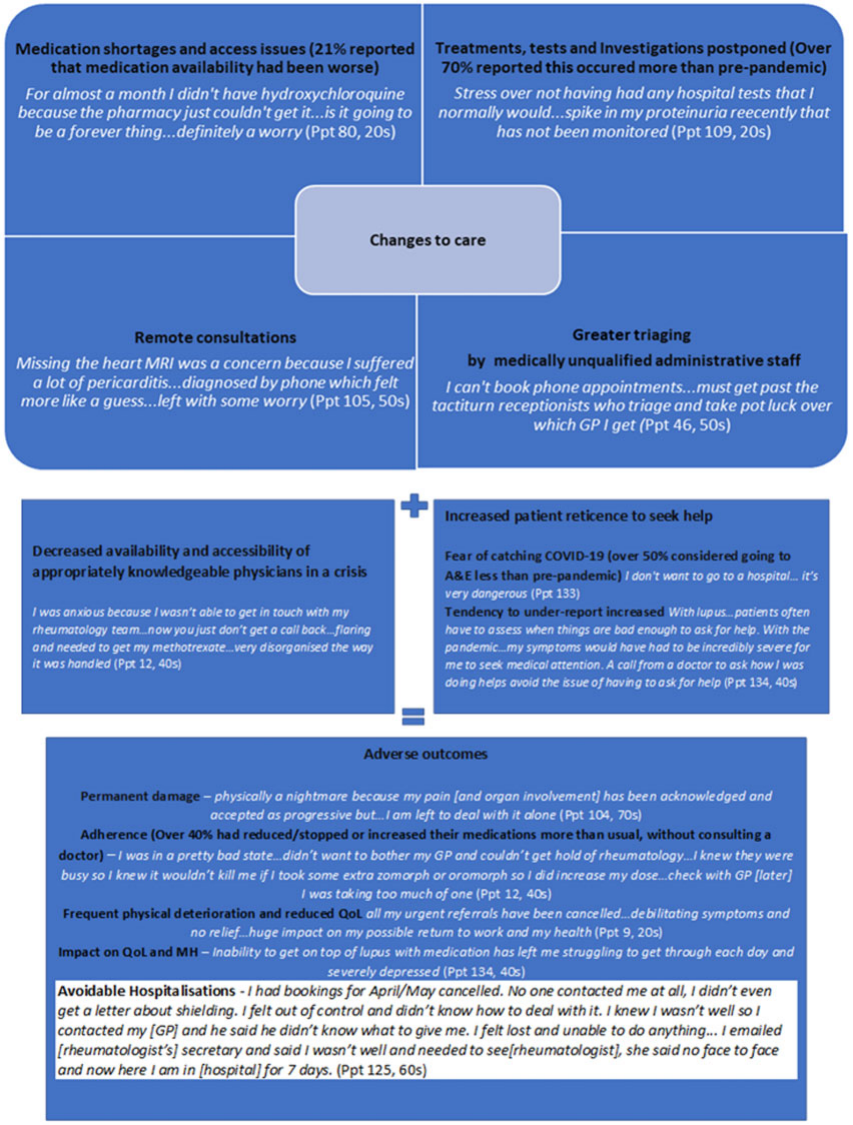
The perfect storm contributing to adverse outcomes

The impact of COVID-19, combined with the changes to care, were exacerbated by decreased physician ability to help and increased patient reticence to seek help, creating the perfect storm for adverse outcomes, as depicted in Fig. 3. In addition to the high proportion of appointments, tests and treatments cancelled, other factors acted against prompt effective treatment, in different combinations for different individuals, including:

- Not having wanted to go to hospital for fear of catching COVID-19 (with 71% agreeing that this applied to them):

I have not reached out for medical attention when I've needed it as I'm so scared I'll be sent to hospital and catch coronavirus. (Participant 133, 20s)

- Not wanting to bother busy physicians (with 68% agreeing):

Recognize the enormity of the task that they’re having to undertake.… I was very concerned of that the whole way through. I hope I don’t get sick and bother a doctor. (Participant 122, 60s)

- Inaccessibility of appropriately knowledgeable physicians owing to the NHS ‘putting all their eggs in the COVID basket’ (Participant 12, 40s) and widespread redeployment of rheumatologists.
- Remote consultations; some positives were identified, especially in terms of convenience: [‘You don’t have to drag yourself somewhere do you?’ (Participant 75, 40s)]; and yet multiple disadvantages were reported, particularly the reduced accuracy of clinical judgement where a visual or hands-on approach was required:

You just want someone to put a hand on it and tell me I’m not over-reacting or under-reacting … they could have got me to hospital earlier [if I’d been seen face-face], but that’s probably my fault as I’m not great at seeking help. (Participant 80, 20s)

- Increased tendency to under-report; many patients had clearly been conditioned in avoidance and symptom under-reporting by difficult diagnostic journeys and negative medical experiences:

I feel like I would rather die than see anyone.… I always feel that [rheumatologists] minimize everything.… I’m now someone that feels a burden a lot of the time. I hate to ask for help. If I ask for help, I need it. (Participant 28, 50s)

### Theme 5: Identifying the patients most vulnerable to a reduction in care

The patient groups identified (in Table 3) as potentially most vulnerable to a reduction in care included those with severe active disease (particularly renal or pulmonary), the newly diagnosed, those who had started new treatments with regular monitoring required immediately before the pandemic, and those with uncertain diagnoses:

I’m on nobody’s list and nobody’s radar … waiting for fuller diagnosis.… My care was bad enough before the pandemic, now it’s going to be even worse … you know they tell you how many people have died of coronavirus but all the other people who have died of other things, that doesn’t matter. (Participant 57, 50s)

**Table 3:** Potentially the most vulnerable groups to the pandemic-initiated changes in health care

**Those on the diagnostic journey** Haven’t been diagnosed with anything yet but getting pretty desperate. GP says there’s nothing they can do until things open back up again.… I’m getting very annoyed seeing everyone on the telly saying the NHS is open when it clearly isn’t.… I guess it’s just a waiting game for me now … though I get the impression getting diagnosed is a bit of a rigmarole for most people regardless of a global pandemic, right? (Forum member, 30s) **The newly diagnosed, awaiting further clarity** Recent diagnosis anyway, and the pandemic has made me hypervigilant, definitely increased my anxiety.… I’d various scans and tests that showed I needed to be investigated and see relevant specialists, so when they were cancelled it was quite anxiety provoking. (Participant 108, 30s) **Patients requiring regular monitoring for potential organ damage, particularly nephritis** I was due to have a kidney test … GPs cancelled … got the ‘Well it’s only routine and just give us a ring if you start feeling ill’. But if I start feeling ill from my kidneys then things have got really bad. (Participant 10, 60s) **Those with active, severe disease requiring urgent multi-disciplinary care** Urgent referrals put in at the start of February, all are now postponed … been hospitalized four times during lockdown … [have had] kidney infections … sepsis … adrenal crisis … vision loss … severe chest pain … regularly losing consciousness … cardiology clinics shut completely … unfortunately, secondary care has basically ground to a halt. (Participant 9, 20s) **Patients with unexplained, unmonitored symptoms pre-pandemic** I just think I should roll over and die. So, if I got coronavirus, it matters that I can’t breathe, but because they can’t find out what’s wrong with me and I’m not on a list somewhere, it doesn’t matter if I can’t breathe and I die tomorrow. (Participant 57, 50s) **The most health-care avoidant owing to traumatic diagnostic journeys** I have often waited and waited to go to the GP as it has taken a long, long time to get diagnosed and spent years feeling like everyone thought I was a hypochondriac, but I honestly knew something wasn’t at all right and was steadily getting worse. (Participant 134, 40s)

GP: general practitioner; NHS: National Health Service.

Those with a longer disease duration might be less vulnerable to temporary reductions in care and generally expressed more confidence in self-management during interviews. There were weak positive correlations between disease duration and feeling they could control their disease (*r* = 0.26, *P* = 0.01) and with feeling they had adapted to life with their disease (*r* = 0.33, *P* = 0.01): ‘learn to be at peace with what you have’ (Participant 104, 70s). However, even the most experienced patients at times require prompt professional medical support to avoid permanent damage, but reports suggest that this was not always forthcoming during the pandemic.

There were no statistically significant differences between demographic groups, including in terms of fears of catching COVID-19 and perceptions of medical support. However, some patients with co-morbidities, greater age and/or belonging to a Black, Asian or minority ethnic (BAME) group mentioned increased stress and more cautious behaviour on receipt of the information of these being additional risk factors:

When this information came to light, I was very alarmed.… On top of my health, I was now one of the people who also fell into the ethnic [risk] groups.… It did cause me a lot of stress, and I had a flare up.… I am very worried about the future. (Participant 132, 40s)

## Discussion

Patients with SARDs do not appear to be at increased risk of contracting COVID-19 [26], and those who do become infected have been found to have a ‘small but significant’ increased risk of death [26], with other chronic conditions, including diabetes, having a far more substantial impact [27]. Consequently, the greater threat to many lupus/SARD patients might not have been COVID-19 itself, but the NHS response in curtailing normal care [9]. In particular, the frequent cancellation of their appointments, problems in accessing medications and reduced monitoring of symptoms might well have increased the risk of long-term damage [28], and possible death in severe cases [29], from not quickly detecting [30] and treating flare-ups, infections, and/or responding to medication side-effects [10].

Consistent with other studies [28], we found that the adverse impact of diverting clinical care from SARD to COVID-19 patients was compounded by health-care avoidance [31] by patients fearful of catching the virus and not wanting to be an additional burden on over-stretched clinicians. However, those who did attend hospital largely reported feeling safer than expected and some had a much quicker and improved experience compared with pre-pandemic visits owing to reduced hospital activity. Inadequate knowledge of SARDs among physicians [11, 32–34] is likely to remain a major problem for these patients, both in terms of feeling medically secure and obtaining the correct treatment, until greater focus is given to these diseases in medical training and research. One potential advantage of the pandemic is the increased medical focus on neuroinflammatory mechanisms [35] and pathological fatigue, experienced by many SARDs patients (and other patient groups, such as multiple sclerosis and myalgic encephalomyelitis/chronic fatigue syndrome patients), which to date have been poorly understood and under-researched.

Our most concerning finding was that numerous, previously responsive, rheumatology departments and physicians were reported not to respond effectively (or not at all, in some cases) to repeated requests for medical advice and help. This was felt to have contributed not only to many patients deteriorating physically, with some requiring hospitalization, but also to a widespread sense of medical abandonment. Abandonment was also a major finding in a recent patient-view survey from the Rare Autoimmune Rheumatic Disease Alliance (RAIRDA) [36]. Conversely, our findings also indicated that physicians who contacted their patients and remained quickly accessible had a positive influence on multiple patient-reported health outcomes. This included a strong positive correlation between patients feeling medically supported during the pandemic and how helpful their doctors were in answering COVID-19 risk questions (*r* = 0.776). This is likely to reflect the more positive communication and care overall from physicians who took the time to give personalized risk information. Unsolicited contact was particularly appreciated and viewed as demonstrating genuine care for the patient at a very anxious time for many. One UK rheumatology department reported that their continuation during the pandemic of an established helpline and clear, prompt communication of individual risks, although an ‘enormous task’, was felt to have provided continuity of care and reduced patient anxiety [9].

Our previous research found that a trusting medical relationship, including reassurance of accessibility in a health crisis, is one of the key components of medical security [14]. The building of trusting medical relationships is likely to have been adversely affected by the pandemic and might be impacted further by the widespread move to video/telephone consultations. Although some benefits of remote consultations were identified, especially in the short term, we have serious concerns regarding the safety and acceptability of a long-term move post-pandemic to more remote consultations [37] for SARD patients. As we have shown previously, SARDs patients can carry a burden of medical insecurity and distrust of clinicians [11, 14]. Without face-to-face consultation, such patients might fail to re-establish the trust needed to discuss their concerns. Furthermore, many features of active SARDs are challenging to diagnose without physical examination and further tests [10].

Highlighting these many negative impacts on care for SARD patients is essential for future planning and does not diminish the great efforts and sacrifices made by many clinicians [38]. COVID-19 has created an unprecedented, stressful and potentially life-threatening situation for clinicians and their patients. Both might have psychological distress and longer-term damage from their experiences, which need to be considered further.

Other studies [4] report that the COVID crisis prompted the cancellation of chronic care services and emphasize the importance of a continuing provision in order to prevent excess non-COVID mortality. Pope [39] argues that worse overall outcomes might have accrued from the diversion of resources, undertreatment, non-adherence and additional stressors causing disease flares in patients with SARDs. Likewise, Tapper & Asrani [40] have warned of longer-term adverse sequelae as a result of the prioritization of COVID-19, including a lengthy period of ‘suboptimal outcomes characterized by missed diagnoses, progressive disease and loss to follow-up’. This seems particularly concerning for SARDs patients, for whom diagnosis is challenging and where early treatment and correct monitoring can reduce disease progression and damage [10].

The results might not be generalizable to the wider SLE and SARD population because participants were from a co-existing trial recruited to measure peer support, which might not have attracted a representative sample, and diagnoses were self-reported. However, study validity was strengthened through triangulating our quantitative and qualitative findings; anomalous case analysis [24], and ‘member checking’ [23] agreement of emerging themes with participants and LUPUS UK forum users. As is commonly used in qualitative research, purposive [41] (purposeful selection to ensure a range of characteristics/opinions rather than random) sampling of interviewees from survey responses ensured a good range of sociodemographic and experience coverage. A limitation is that we were unable to obtain many views from male patients. Although they are in the minority with lupus (5–10% of cases), their views and experiences might differ. Ethnicity was more representative among interview participants owing to purposive sampling, but sign-up to the study was proportionally lower in BAME groups, and this might have skewed the results. Bearing in mind that both SLE [10] and COVID-19 [42] have a disproportionate impact on minority ethnic communities, there is a pressing need to prevent ‘widening disparities among patients with rheumatic diseases in the COVID-19 era’ [43].

In conclusion, it is essential that future pandemic planning facilitates appropriate, prompt care for all SARD patients with clear and accessible lines of communication. This will reduce the short- and long-term costs (personal, NHS and societal) identified from inadequate routine and emergency care during the pandemic for this group of patients, many of whom have life-changing and/or life-threatening disease. Future plans should consider the needs of particularly vulnerable subgroups.

## Supplementary Material

rkaa072_Supplementary_Data

## References

[1] Anandaciva S , Ewbank L, Thompson J, McKenna H. NHS hospital bed numbers: past, present, future. London: Kings Fund, 2020 . https://www.kingsfund.org.uk/publications/nhs-hospital-bed-numbers (6 July 2020, date last accessed).

[2] Clift AK. Anatomising failure: there should be a statutory public inquiry into the UK Government’s handling of COVID-19. J R Soc Med 2020;113:230–1. 32521194 10.1177/0141076820925778 PMC7439592

[3] Moss P , Barlow G, Easom N, Lillie P, Samson A. Lessons for managing high-consequence infections from first COVID-19 cases in the UK. Lancet 2020;395:E46. 32113507 10.1016/S0140-6736(20)30463-3 PMC7133597

[4] Coronini-Cronberg S , Maile EJ, Majeed A. Health inequalities: the hidden cost of COVID-19 in NHS hospital trusts? J R Soc Med 2020;113:179–84. DOI: 10.1177/0141076820925230 32407644 10.1177/0141076820925230 PMC7366335

[5] Majeed A , Maile EJ, Bindman AB. The primary care response to COVID-19 in England’s National Health Service. J R Soc Med 2020;113:208–10. 32521196 10.1177/0141076820931452 PMC7439588

[6] Beran D , Perone SA, Perolini MC et al Beyond the virus: ensuring continuity of care for people with diabetes during COVID-19. Prim Care Diabetes 2020;15:16–17.doi: 10.1016/j.pcd.2020.05.014. Epub 2020 May 30, S1751-9918(20)30199-6. 32535088 10.1016/j.pcd.2020.05.014 PMC7260491

[7] Dacre J. Virtual rheumatology during COVID-19: a personal perspective. Rheumatol Ther 2020;7:429–31. 32725408 10.1007/s40744-020-00224-5 PMC7385474

[8] Henderson LA , Canna SW, Schulert GS et al On the alert for cytokine storm: immunopathology in COVID‐19. Arthritis Rheumatol 2020;72:1059–63. 32293098 10.1002/art.41285 PMC7262347

[9] Nune A , Iyengar K, Ahmed A, Sapkota H. Challenges in delivering rheumatology care during COVID-19 pandemic. Clin Rheumatol 2020;39:2817–21. 32712743 10.1007/s10067-020-05312-z PMC7382561

[10] Gordon C , Amissah-Arthur M-B, Gayed M et al The British Society for Rheumatology guideline for the management of systemic lupus erythematosus in adults. Rheumatology 2018;57:e1–e45. 29029350 10.1093/rheumatology/kex286

[11] Sloan M , Harwood R, Sutton S et al Medically explained symptoms: a mixed methods study of diagnostic, symptom and support experiences of patients with lupus and related systemic autoimmune diseases. Rheumatol Advan Pract 2020:4:rkaa006 10.1093/rap/rkaa006 32373774 PMC7197794

[12] Tunnicliffe DJ , Singh‐Grewal D, Craig JC et al Perspectives of medical specialists from different disciplines on the management of systemic lupus erythematosus: an interview study. Arthritis Care Res 2018;70:1284–93. 10.1002/acr.23469 29136338

[13] Romão VC , Cordeiro I, Macieira C et al Rheumatology practice amidst the COVID-19 pandemic: a pragmatic view. RMD Open 2020;6:e001314. 32584782 10.1136/rmdopen-2020-001314 PMC7425193

[14] Sloan M , Naughton F, Harwood R et al Is it me? The impact of patient–physician interactions on lupus patients’ psychological well-being, cognition and health-care-seeking behaviour. Rheumatol Advan Pract 2020;rkaa037 10.1093/rap/rkaa037 32974426 PMC7498933

[15] Gianfrancesco M , Hyrich KL, Al-Adely S et al Characteristics associated with hospitalisation for COVID-19 in people with rheumatic disease: data from the COVID-19 Global Rheumatology Alliance physician-reported registry. Ann Rheum Dis 2020;79:859–66. 32471903 10.1136/annrheumdis-2020-217871 PMC7299648

[16] Mendel A , Bernatsky S, Askanase A et al Hydroxychloroquine shortages among patients with systemic lupus erythematosus during the COVID-19 pandemic: experience of the Systemic Lupus International Collaborating Clinics. Ann Rheum Dis 2020;Published on 25 June 2020, doi: 10.1136/annrheumdis-2020-218164 10.1136/annrheumdis-2020-218164 32586918

[17] Jakhar D , Kaur I. Potential of chloroquine and hydroxychloroquine to treat COVID-19 causes fears of shortages among people with systemic lupus erythematosus. Nat Med 2020;26:632. 10.1038/s41591-020-0853-0 32269358

[18] Rutter M , Lanyon P, Sandhu R et al Estimation of the burden of shielding among a cross-section of patients attending rheumatology clinics with SLE—data from the BSR audit of systemic lupus erythematosus. Rheumatology 2020;keaa620. 10.1093/rheumatology/keaa620 PMC7665698 33677595

[19] ECDCP (European Centre for Disease Prevention and Control). Rapid risk assessment: coronavirus disease 2019 (COVID-19) in the EU/EEA and the UK – eleventh update: resurgence of cases. 2020 . https://www.ecdc.europa.eu/en/publications-data/rapid-risk-assessment-coronavirus-disease-2019-covid-19-eueea-and-uk-eleventh (6 July 2020, date last accessed).

[20] Cresswell J , Clark V. Designing and conducting mixed methods research. London, UK: Sage, 2017.

[21] Tennant R , Hiller L, Fishwick R et al The Warwick-Edinburgh Mental Well-being Scale (WEMWBS): development and UK validation. Health Qual Life Outcomes 2007;5:63 10.1186/1477-7525-5-63 18042300 PMC2222612

[22] Braun V , Clarke V. Using thematic analysis in psychology. Qual Res Psychol 2006;3:77–101. DOI: 10.1191/1478088706qp063oa

[23] Birt L , Scott S, Cavers D, Campbell C, Walter F. Member checking: a tool to enhance trustworthiness or merely a nod to validation? Qual Health Res 2016;26:1802–11. 27340178 10.1177/1049732316654870

[24] Pearce LD. Integrating survey and ethnographic methods for systematic anomalous case analysis. Sociol Methodol 2002;32:103–32.

[25] Tong A , Sainsbury P, Craig J. Consolidated criteria for reporting qualitative research (COREQ): a 32 item checklist for interviews and focus groups. Int J Qual Health Care 2007;19:349–57. 17872937 10.1093/intqhc/mzm042

[26] Grange L , Guilpain P, Truchetet M-E, Cracowski J-L. Challenges of autoimmune rheumatic disease treatment during the COVID-19 pandemic: a review. Therapies 2020;75:335–42. 10.1016/j.therap.2020.06.013 PMC7320663 32665090

[27] Kumar A , Arora A, Sharma P et al Is diabetes mellitus associated with mortality and severity of COVID-19? A meta-analysis. Diabetes Metab Syndr 2020;14:535–45. 32408118 10.1016/j.dsx.2020.04.044 PMC7200339

[28] Ugarte-Gil MF , Acevedo-Vásquez E, Alarcón GS et al The number of flares patients experience impacts on damage accrual in systemic lupus erythematosus: data from a multiethnic Latin American cohort. Ann Rheum Dis 2015;74:1019–23. 24525909 10.1136/annrheumdis-2013-204620

[29] Whitelaw DA , Gopal R, Freeman V. Survival of patients with SLE admitted to an intensive care unit—a retrospective study. Clin Rheumatol 2005;24:223–227. 15565499 10.1007/s10067-004-1007-3

[30] Horisberger A , Moi L, Ribi C, Comte D. Impact of COVID-19 pandemic on SLE: beyond the risk of infection. Lupus Sci Med 2020;7:e000408. 32376774 10.1136/lupus-2020-000408 PMC7223264

[31] Ciacchini B , Tonioli F, Marciano C et al Reluctance to seek pediatric care during the COVID-19 pandemic and the risks of delayed diagnosis. Ital J Pediatr 2020;46:87. 32600464 10.1186/s13052-020-00849-w PMC7322712

[32] Day C , Yeh A, Franco O, Ramirez M, Krupat E. Musculoskeletal medicine: an assessment of the attitudes and knowledge of medical students at Harvard medical school. Acad Med 2007;82:452–7. 17457065 10.1097/ACM.0b013e31803ea860

[33] Akesson K , Dreinhöfer K, Woolf AD. Improved education in musculoskeletal conditions is necessary for all doctors. Bull World Health Organ 2003;81:677–83. 14710510 PMC2572534

[34] Gamez-Nava JI , Gonzalez-Lopez L, Davis P, Suarez-Almazor ME. Referral and diagnosis of common rheumatic diseases by primary care physician. Br J Rheumatol 1998;37:1215–9. 9851272 10.1093/rheumatology/37.11.1215

[35] Debnath M , Berk M, Maes M. Changing dynamics of psychoneuroimmunology during the COVID-19 pandemic. Brain Behav Immun Health 2020;5:100096.doi: 10.1016/j.bbih.2020.100096. 32566934 10.1016/j.bbih.2020.100096 PMC7295528

[36] RAIRDA. Chronic crisis. The impact of COVID-19 on people with rare autoimmune rheumatic diseases. The Rare Autoimmune Rheumatic Disease Alliance. https://rairdaorg.files.wordpress.com/2020/08/chronic-crisis-report-june-2020-1.pdf.

[37] Bhaskar R , Nowak RJ, Roda R et al Teleneurology during the COVID-19 pandemic: a step forward in modernizing medical care. J Neurol Sci 2020;414:116930. 32460041 10.1016/j.jns.2020.116930 PMC7241381

[38] Simons J , Vaughan J. Sacrifice and risk in the time of COVID-19. Future Healthc J 2020;7:158–60. 32550647 10.7861/fhj.2020-0035 PMC7296565

[39] Pope JE. What does the COVID-19 pandemic mean for rheumatology patients? Curr Treatm Opt Rheumatol 2020;30:1–4. 10.1007/s40674-020-00145-y PMC7191545 32355607

[40] Tapper EB , Asrani SK. The COVID-19 pandemic will have a long-lasting impact on the quality of cirrhosis care. J Hepatol 2020;73:441–5. 32298769 10.1016/j.jhep.2020.04.005 PMC7194911

[41] Lavrakas PJ. Encyclopedia of survey research methods. Vols. 1–0. Thousand Oaks, CA: Sage, 2008: doi: 10.4135/9781412963947

[42] Kirby T. Evidence mounts on the disproportionate effect of COVID-19 on ethnic minorities. Lancet Respir Med 2020;8:547–8. 32401711 10.1016/S2213-2600(20)30228-9 PMC7211498

[43] Feldman CH , Ramsey‐Goldman R. Widening disparities among patients with rheumatic diseases in the COVID‐19 era: an urgent call to action. Arthritis Rheumatol 2020;72:1409–11. 32379381 10.1002/art.41306 PMC7267415

