# Supplementary material for: The impact of the COVID-19 pandemic on the medical care and health-care behaviour of patients with lupus and other systemic autoimmune diseases: a mixed methods longitudinal study
Source: Rheumatol Adv Pract. 2020 Dec 14;5(1):rkaa072. doi: 10.1093/rap/rkaa072 (PMC7798562; doi:10.1093/rap/rkaa072)
Supplement: rkaa072_Supplementary_Data [file rkaa072_supplementary_data.docx]

Supplementary Data S1: Interview schedule


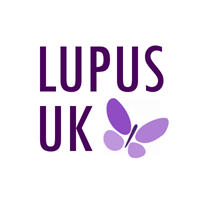

**Interview schedule**

Semi-structured. Additional individually tailored questions based on survey responses. Follow up questions and probes adapted for each individual participant depending on their experiences and priorities.

**Section 1. Pre-pandemic - Usual care and relationships with clinicians, wider society**

- How long with the disease, level of adaptation and acceptance.
- Main symptoms now, severity and regularity of flares
- Positives/negatives of usual care pre-pandemic. Regularity of appointments
- Positives/ negatives of relationships and support from family and friends (+ work if employed)

**Section 2. Feelings/ experiences at start of pandemic**

- Views on risks
- Initial communications from NHS, Gov, clinicians about individual risks (were they in shielded group, who communicated risk level, clarity, how quickly, how felt to be included/not included, did a clinician make contact and explain/offer support)
- Impacts, changes made to lifestyle, feelings, fears. Any further changes over the period of lockdown.

**Section 3. Medical care and support during pandemic**

- Reasons for seeking/not seeking help. Level of help provided
- Appointments differed, cancellations, blood monitoring/ tests etc, remote consultations – positives/negatives
- Ease of access, feelings of being supported/unsupported
- Issues of attending appointments/ any A&E visits – quality of care, fears of catching virus
- Treatment issues, medication supply, adherence
- How feel COVID and response impacted chronic disease care overall, changes over course of pandemic, concerns for future care

**Section 4. Impact of COVID-19 and changes in medical care on Mental health and wellbeing**

- Challenges, stresses faced, any positives
- How feel coped mentally in pandemic so far. Coping strategies. Changes over time.
- Discuss MH – views on causes (and exacerbators/mitigators). Formal or self-help sought
- How changes to medical care influenced mental (and physical) health - +ves and -ves

**Section 5. Summary**

Summarise, clarify and probe any points further

What’s most important to this participant

Anything further

Supplementary Data S2: COREQ (COnsolidated criteria for REporting Qualitative research) Checklist^20^ and further details of methods

| Topic | Item No | Guide Questions/Description | Details and/or Reported on Page No. |
| --- | --- | --- | --- |
| **Domain 1: Research team and reflexivity** |  |  |  |
| *Personal characteristics* |  |  |  |
| Interviewer/facilitator | 1 | Which author/s conducted the interview or focus group? | MS |
| Credentials | 2 | What were the researcher’s credentials? | Supplementary Information |
| Occupation | 3 | What was their occupation at the time of the study? | Research Associate  Page 5 + Supplementary Information |
| Gender | 4 | Was the researcher male or female? | F, page 5 |
| Experience and training | 5 | What experience or training did the researcher have? | Supplementary Information |
| Relationship established | 6 | Was a relationship established prior to study commencement? | Supplementary Information |
| Participant knowledge of the interviewer | 7 | What did the participants know about the researcher? | Supplementary Information |
| Interviewer characteristics | 8 | What characteristics were reported about the interviewer/facilitator? | Page 5 + Supplementary Information |
| **Domain 2: Study design** |  |  |  |
| *Theoretical framework* |  |  |  |
| Methodological orientation and Theory | 9 | What methodological orientation was stated to underpin the study? | Supplementary information |
| *Participant selection* |  |  |  |
| Sampling | 10 | How were participants selected? | Page 4 + Supplementary Information |
| Method of approach | 11 | How were participants approached | Email  Supplementary Information |
| Sample size | 12 | How many participants were in the study | 111 for survey and 28 for interview  Page 4 + supplementary information |
| Non-participation | 13 | How many people refused to participate or dropped out? | None refused but several uncontactable.  Supplementary Information |
| *Setting* |  |  |  |
| Setting of data collection | 14 | Where was the data collected? | Supplementary Information |
| Presence of non-participants | 15 | Was anyone else present besides the participants and researchers? | Supplementary Information |
| Description of sample | 16 | What are the important characteristics of the sample? | Page 7 + Table 1 + supplementary information |
| *Data collection* |  |  |  |
| Interview guide | 17 | Were questions, prompts, guides provided by the authors? | Yes. Supplementary Information (Guide at supplementary information 1) |
| Repeat interviews | 18 | Were repeat interviews carried out? | Supplementary Information |
| Audio/visual recording | 19 | Did the research use audio or visual recording to collect the data? | Audio. Supplementary information |
| Field notes | 20 | Were ﬁeld notes made during and/or after the interview or focus group? | Yes. Supplementary Information |
| Duration | 21 | What was the duration of the inter views or focus group? | Approx 60 mins each. Supplementary information |
| Data saturation | 22 | Was data saturation discussed | Yes. Pg 5+ supplementary information |
| Transcripts returned | 23 | Were transcripts returned to participants for comment and/or correction? | No |
| **Domain 3: analysis and findings** |  |  |  |
| *Data analysis* |  |  |  |
| Number of data coders | 24 | How many data coders coded the data? | Two. Pg 4 + supplementary information |
| Description of the coding tree | 25 | Did authors provide a description of the coding tree? | Available on request |
| Derivation of themes | 26 | Were themes identiﬁed in advance or derived from the data? | Derived from data. Supplementary information |
| Software | 27 | What software, if applicable, was used to manage the data? | Nvivo 12. Supplementary information |
| Participant checking | 28 | Did participants provide feedback on the ﬁndings? | Yes. Supplementary information and discussion |
| *Reporting* |  |  |  |
| Quotations presented | 29 | Were participant quotations presented to illustrate the themes/ﬁndings? | Yes. |
| Data and findings consistent | 30 | Was there consistency between the data presented and the ﬁndings? | Yes. |
| Clarity of major themes | 31 | Were major themes clearly presented in the ﬁndings? | Yes. |
| Clarity of minor themes | 32 | Is there a description of diverse cases or discussion of minor themes? | Yes. |

**Domain 1: Research team and reflexivity**

This research was conducted by a multi-disciplinary research team, incorporating behavioural scientists, patients, statisticians, LUPUS UK staff and rheumatologists, several of whom were involved in the direct care of both COVID-19 and rheumatology patients during the pandemic. Different viewpoints were shared; and team members were aware of the potential for bias arising from their own experiences. All interviews were carried out by MS, a qualitatively trained, female research associate with many years of experience in interviewing different patient groups; and 25% of interviews were double-coded by RH, a male, socio-legal researcher. Both of these researchers have a systemic autoimmune rheumatic disease (SARD) (as do approx. 50% of the research team) which appeared to facilitate rapport with study participants and assist in understanding their experiences.

A strength of this study team is its in-depth knowledge of SARDs, with the study rheumatologists having over 60 years combined experience working with patients with SARDs and the patient representatives and patient-researchers together having over 100 years experience of having SARDs. LUPUS UK staff were on the frontline responding to a deluge of phone calls and forum posts from often distressed patients during the pandemic; and also added their perspective from many years of assisting and representing patients.

Some of the research team are well-known to patients who use the LUPUS UK forum. Emerging study findings are shared and discussed in the public forum, so as to strengthen validity and help ensure that the study gives “voice” to community views; and the final published papers are made available (through journal links) on the forum and summaries provided in the Lupus UK magazine. The research also aimed to further the interests of the researched communities^43^ through promoting improvements to care.

**Domain 2: Study design**

This was a mixed methods study, analysing quantitative changes from a survey completed between 4-10 March 2020, which approx. 2 weeks prior to the UK lockdown and just prior to COVID-19 being declared a pandemic on 11 March 2020, and comparing results with the follow-up survey completed (10-21 June) during the pandemic. Qualitative data was collected from multiple sources, using a range of methods, including: open-ended questions on the follow-up survey, ethnographic immersion in the forum by researchers and patient representatives, thematic analysis of pandemic related forum conversations and participant email conversations from March-July 2020, and in-depth interviews with 28 purposively selected study participants.

Study participants were those who were initially recruited for a randomised controlled intervention trial to determine the feasibility, effectiveness and acceptability of autoimmune patient peer support by email group. One hundred and thirty-nine participants were recruited for the original peer support trial by open invitation to all who fitted the eligibility criteria through the LUPUS UK online forum and the lupus support UK Facebook group. They were randomly (varying block sizes) allocated to one of three equally-sized groups: control, peer support email group, peer support email group + research involvement. The two peer support groups were divided into smaller sub-groups of 5/6 participants to form email support groups. In addition, the research involvement groups (8 groups of approx. 6 participants) were asked to discuss between themselves and with the lead researcher various research questions, which were sent to the group approximately every 7-14 days. Due to the pandemic being of most immediate concern to participants during this initial follow-up period, the majority of conversations were about their pandemic-induced reactions and feelings, including many discussions on the differing provision of medical care received by participants.

Baseline and follow-up questionnaires were originally designed to assess various measures of wellbeing, mental health (MH) and satisfaction with medical care. Various validated tools were used to measure changes in wellbeing, disease adaptability and illness impact including: The Warwick-Edinburgh Mental Wellbeing Scale (WEMWS)^19.^ Multiple patients also assisted in designing questions. Due to the pandemic, the follow-up questionnaires had a substantial number of additional questions in order to also measure changes in care, healthcare-behaviours and emotions generated by the pandemic. These questionnaires were completed between 10th and 21st June. Up to three reminders were sent to non-responders. Second follow-up is due at one-year post baseline (March 2021). The questionnaire also contained open-ended questions to ascertain views on the pandemic, changes to MH and care, and views on the government and NHS response.

Interview participants were then purposively selected from survey response to ensure the full range of experiences of pandemic medical care and attitudes towards their care. Selection occurred to ensure as representative a range as possible of disease and socio-demographic characteristics. This was achieved in all areas except for gender, due to only two males signing up for the original study and both being unresponsive to requests for interview. Potential interviewees were initially approached by the email contact details provided on the survey. Up to three emails were sent for non-responders. No potential participants declined the invitation to participate, although five did not respond to emails, and two initially responded to agree to interview then were subsequently uncontactable.

Due to the pandemic and varied geographical locations of participants, interviews were conducted largely on the telephone. Several interviews were carried out by email when that was the participant’s preference. These were found to generate equally in-depth responses, especially following probing questions to the initial responses. Following saturation, four additional short interviews were undertaken to confirm patient agreement with emerging themes.

The interview schedule was designed following consultation with multiple patients, researchers, LUPUS UK and rheumatologists. It contained standard questions and tailored questions for each individual participant based on their survey responses. Interviews were semi-structured, with additions following earlier stages of analysis and interviews. They were gently guided by the interviewer to answer the study aims, but also with flexibility to explore in-depth each participant’s priorities and interests.

**Domain 3: analysis and findings**

Quantitative and qualitative methods were mixed at every stage from design through collection, to analysis and presentation in a multi-stage design. The results from one method were compared and tested against the other, in order to more deeply understand the findings, and mitigate the respective weaknesses of each methodology if used individually^20^. The qualitative components were both exploratory and explanatory at different time points. The forum analysis and conversations with the research involvement study participants were largely exploratory to inform the development of the follow-up survey. The interviews were conducted in July 2020, after analysis of the quantitative data.

Analysis of quantitative data was aided by SPSS V26, predominantly using comparisons of means at baseline and follow-up, and correlations between various measures, using Pearson’s correlation coefficient. The use of the WEMWS as a primary outcome for the interventional study was agreed in the statistical analysis plan prior to data collection. The scale has 14 questions, scoring 1-5 with a lower score being negative. Questions include confidence, optimistic for future, thinking clearly etc. The lowest score possible for someone who feels/is doing those things ‘none of the time’ is 14.

As there was no significant difference found between control and intervention groups for the original peer support study, we were able to combine all participants who had completed both baseline and follow-up questionnaires (111/139 – 80% response rate) into one group for this additional study to explore the impact of COVID-19 on medical care.

Quantitative analysis for this study used Pearson’s correlation coefficient. Correlation (r) demonstrates the degree and direction of the relationship between two variables with +1 and -1 being a perfect positive/negative correlation and 0 being no linear relationship. The p-value indicates the level of statistical significance (P values of 0.05 and less are considered to be statistically significant – as in this finding is unlikely to occur by chance). A negative correlation, for example ‘feeling medically supported’ with ‘level of fatigue’ at -0.265, shows a weak inverse relationship, meaning that as level of feeling medically supported increases, level of reported fatigue decreases.

Qualitative analysis was thematic, with data management aided by NVivo 12. Themes were directly generated from the interview data, discussed among the team and with patients, and then developed in subsequent interviews using the constant comparative method. Interviews continued until a high level of theoretical saturation was achieved. This was when additional interviews did not provide any additional insights.

The stages of analysis (some stages were ongoing or cyclical rather than sequential) involved:

1. Immersion in the forum - MS, LUPUS UK staff and patient representatives read posts relating to the pandemic and discussed emerging group views and issues related to the aims of this paper. A more formal period of thematic analysis was then used to both inform questions for the follow-up survey and to triangulate findings from the other qualitative methods.
2. Participants in the intervention research involvement groups (N=40) were invited to discuss their views and experiences of their changing care and health related to the pandemic, with their email groups and MS, approx. every 7-14 days, in the 12 weeks between baseline and first follow-up questionnaire.
3. The exploratory stages listed as 1 and 2, were used in conjunction with input from rheumatologists and LUPUS UK, to determine the pandemic-specific questions for the follow-up survey and interviews.
4. The quantitative results from the survey were analysed using SPSS, graphically presented and discussed within the team. The qualitative data from the surveys was combined with the forum analysis and the discussions listed in stages 1 and 2, and a preliminary coding frame developed. Interview questions were formulated from these initial stages, and with further rheumatology, patient and psychology input.
5. Once interviews commenced, data was coded according to the initial coding frame. Several adaptations to the coding frame were necessary as new data and potential themes arose. Each line of each interview, email correspondence or forum conversation relevant to the study questions were coded by MS, using NVivo.
6. Immersion in transcripts and audio recordings continued throughout the interviewing, analysis and writing process with regular re-reading of each transcript/ communication throughout.
7. Double-coding was undertaken by RH who coded 25% of interviews to ensure reliability of the coding frame.
8. Extracts were then combined using NVivo 12. The raw data and the combined coded data were then repeatedly reviewed for emerging themes. Initial ideas for emerging themes were discussed at length and presented to EL, DD’C and CG, who had read multiple transcripts each.
9. Common themes and emerging concepts were then discussed, refined, and agreed by the team.

Themes were constructed from the data, and co-constructed with study participants^44^ and following research team discussions. However, the researchers’ experiences of the pandemic as physicians, patients, advocates and psychologists identified some initial concepts to investigate thus increasing the ‘theoretical sensitivity’^45^. With regards to the quantitative data, MP, the study statistician performed robust checks, and found no bias between characteristics of non-responders/responders with the exception of a higher follow-up rate among younger (<30 years) participants.
